# The MINUTES bundle for the initial 30 min management of undifferentiated circulatory shock: an expert opinion

**DOI:** 10.1186/s12245-024-00660-y

**Published:** 2024-07-25

**Authors:** Ahmed Hasanin, Filippo Sanfilippo, Martin W Dünser, Hassan M Ahmed, Laurent Zieleskiewicz, Sheila Nainan Myatra, Maha Mostafa

**Affiliations:** 1 Department of Anesthesia and Critical Care Medicine, Faculty of Medicine, Cairo University, Cairo, Egypt; 2 University Hospital Policlinico, G. Rodolico - San Marco, Catania, Italy; 3 Department of Surgery and Medical-Surgical Specialties, University of Catania, Catania, Italy; 4 Department of Anaesthesiology and Intensive Care Medicine, Kepler University Hospital and Johannes Kepler University, Krankenhausstrasse 9, Linz, Austria; 5 Leeds Teaching Hospitals NHS Trust, Leeds, UK; 6 Service d’anesthésie réanimation hôpital nord Marseille APHM, C2VN Aix Marseille Université, Marseille, France; 7 Department of Anaesthesiology, Critical Care and Pain, Tata Memorial Hospital, Homi Bhabha National University, Mumbai, India

**Keywords:** Shock, Ultrasound, Vasopressors, Fluids, Norepinephrine, Hypotension, Congestion

## Abstract

Acute circulatory shock is a life-threatening emergency requiring an efficient and timely management plan, which varies according to shock etiology and pathophysiology. Specific guidelines have been developed for each type of shock; however, there is a need for a clear timeline to promptly implement initial life-saving interventions during the early phase of shock recognition and management. A simple, easily memorable bundle of interventions could facilitate standardized management with clear targets and specified timeline. The authors propose the “MINUTES” acronym which summarizes essential interventions which should be performed within the first 30 min following shock recognition. All the interventions in the MINUTES bundle are suitable for any patient with undifferentiated shock. In addition to the acronym, we suggest a timeline for each step, balancing the feasibility and urgency of each intervention. The MINUTES acronym includes seven sequential steps which should be performed in the first 30 min following shock recognition: **M**aintain “ABCs”, **IN**fuse vasopressors and/or fluids (to support hemodynamic/perfusion) and **IN**vestigate with simple blood tests, **U**ltrasound to detect the type of shock, **T**reat the underlying **E**tiology, and **S**tabilize organ perfusion.

## Background

Acute circulatory shock is a life-threatening and highly time-sensitive emergency [1]. For its acute nature, circulatory shock is usually managed by frontline physicians, who are, sometimes, of limited knowledge and experience. Moreover, they may work in limited resource settings. Hence, having a clear, timely, easily memorizable approach may facilitate their performance and finally improve patient’s management.

According to the type of shock, clinical guidelines have been developed and are regularly updated to optimize patient’s management. Once the specific diagnosis of shock has been made, evidence-based management for each type (e.g., septic, hemorrhagic, cardiogenic shock) is well established by international guidelines [2–6]. However, it is critical to minimize the time between shock recognition and initiation of shock- or disease-specific therapies. Currently, specific recommendations for the management of shock due to any etiology in the initial few minutes are lacking.

All physicians, especially junior staff, may benefit from a common pathway to manage patients with undifferentiated shock during the first minutes. Such a common pathway should be applicable to all patients, independently of what type of shock is subsequently diagnosed. In case of rapid clinical deterioration, it is widely accepted that provision of basic and advanced life support must be the first priority [7]. However, it is unclear how these first steps should be implemented, specifically regarding their timing and order.

The aim of this review is to propose a simplified bundle for the initial management of patients with undifferentiated shock. Based on pathophysiological knowledge and scientific evidence, the expert panel suggests an acronym that could help acute care physicians in this task by applying a bundle of sequential interventions. The acronym “MINUTES” was intentionally selected to summarize not only the most important and initial actions but also to focus on the importance of a timely and sequential approach for the main supportive and diagnostic steps. We believe that under most circumstances, the bundle can be accomplished within the first 30 min after shock recognition.

## Do the current guidelines cover the early phase of shock adequately?

Despite the presence of dedicated guidelines for several types of shock, most of these represent recommendations in separate statements without specific order of interventions nor a timeframe for achieving each management step [2–6]. For instance, the Surviving Sepsis Campaign considers several interventions according to different sections (hemodynamic, infection, initial resuscitation, etc.), but a timely approach is not present for all interventions. Moreover, actions should be undertaken to stabilize the patient and decrease mortality and morbidity before the cause of shock is recognized.

Indeed, several guidelines focus on the management of the cause of shock. A summary of the existing guidelines for different types of circulatory shock and the recommendations of its initial management is shown in Table 1. Considering the identified potential gap in clinically-oriented guidelines for the initial management of undifferentiated shock, the expert panel thinks that more attention is required towards common major supportive steps. We think that the proposed first management steps should be implemented independently from the cause of shock and before diagnosis is made, in order to reduce the period of “under-perfusion” and organ damage. Notably, the expert panel also suggests a timeframe for the accomplishment of each phase.

**Table 1:** Guidelines for different types of circulatory shock and recommendations to be implemented within the first 30 min

	Latest guidelines	Recommendations to be implemented within the first 30–60 min
Septic shock	Surviving sepsis campaign 2021 [2]	Measure lactate levels. Obtain blood cultures before administering antibiotics. Administer broad-spectrum antibiotics. Begin to rapidly administer 30 ml/kg crystalloid for hypotension or lactate ≥ 4 mmol/L.
Cardiogenic shock	American Heart Association 2022 [3]	No specific timeline recommendations
Hypovolemic hemorrhagic shock	European Society of Anaesthesiology 2023 [4, 6]	Control any external bleeding and maintain SBP < 90 mmHg (higher target in patients with brain trauma) until bleeding is controlled
Hypovolemic non-hemorrhagic shock	No guidelines identified	
**Obstructive (pulmonary embolism)**	European Society of Cardiology 2019 [5]	No specific timeline guidelines except of urgent echocardiography to detect RV failure for possible reperfusion.
Obstructive (cardiac tamponade)	No guidelines identified	
Obstructive (tension pneumothorax)	No guidelines identified	

MAP: mean arterial pressure, RV: right ventricle, SBP: systolic arterial blood pressure

## The rationale of prioritizing an intervention over other in the bundle

The initial management of patients with undifferentiated shock needs a clear timeline with a pragmatic approach focusing on both the urgency of the interventions as well as the feasibility of such interventions within given time frames. For example, emergency physicians, intensivists, and anesthesiologists who care for patients with undifferentiated shock face the challenge of balancing two key priorities: (1) the need to treat the underlying cause of shock (etiological management); and (2) the need to rapidly restore organ perfusion (pathophysiological management) [8]. Scientific evidence underlines the time sensitivity of the latter intervention (restoration of vital and systemic organ perfusion). Indeed, every additional minute of hypotension is associated with poor outcomes [9]. Moreover, certain causes of shock may require longer time to achieve diagnosis, hence it would be harmful to delay hemodynamic support until the etiology has been clarified. Finally, in the vast majority of patients with shock, early initiation of vasopressors is needed, and in most cases their potential harm is limited. Therefore, the expert panel suggests that vasopressors and/or fluids should be infused early in the management, after basic and advanced life support has been implemented. Of course, such clinical decision should be undertaken only after certain etiologies (e.g., tension pneumothorax), which are rapidly fatal, easily detectable without imaging, and reversible (e.g., decompression), have been ruled out.

The second consideration we took in order to justify the priorities in the acronym was the feasibility and time needed for each intervention. We believe that all supportive and diagnostic tasks should be done whenever possible; however, simple blood investigations (as venous blood gases [VBG]) were prioritized for being easier to obtain and would provide answers within a couple of minutes only.

For the above reasons, we suggest that the logical approach and timeline for the early management of patients with undifferentiated shock should always start with basic and advanced life support, ensuring instant management of rapidly fatal conditions such as massive external bleeding or tension pneumothorax. Restoration of adequate arterial blood pressure levels to ensure vital organ perfusion using vasopressors and/or fluids, as well as performing simple tests like an electrocardiogram or a VBG should be the essential subsequent steps before implementing point-of-care ultrasound to identify the type of shock.

## Components of the MUNITES bundle

**Fig. 1:**
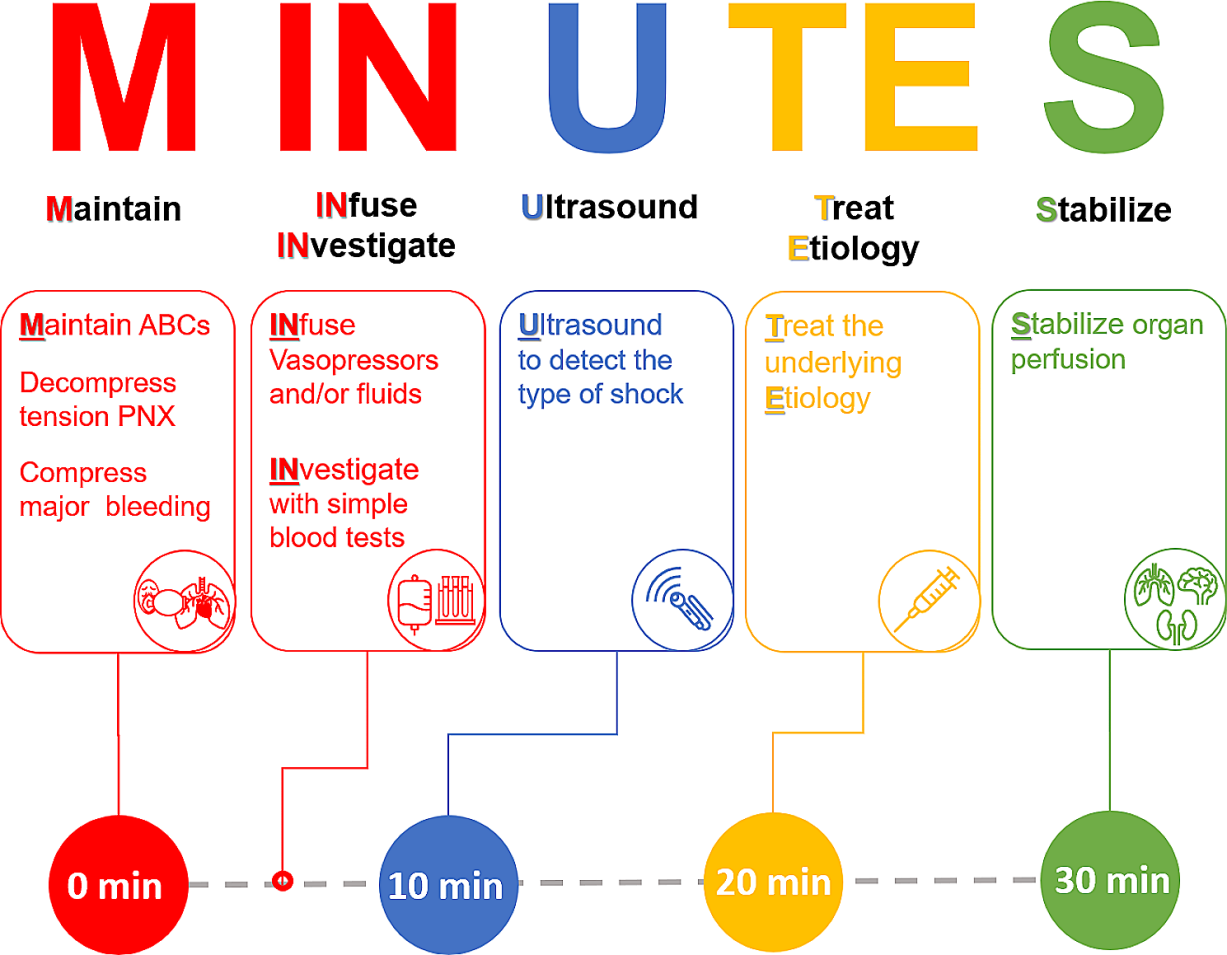
Description of the MINUTES acronym. PNX: pneumothorax

The First-MINUTES bundle includes six sequential steps: **M**aintain “ABCs”, **IN**fuse vasopressors and/or fluids, **IN**vestigate, **U**ltrasound, **T**reat **E**tiology, and **S**tabilize. The First-MINUTES bundle is summarized in Table 2, and graphically displayed in Fig. 1. In the subsequent manuscript, rationale behind each step with its associated time frame is explained.

**Table 2:** The MINUTES acronym to guide the initial management of undifferentiated shock

Letters of the acronym	Description of the item	Objectives
**M**aintain “ABCs”	Provide basic and advanced life support (e.g., compress external bleeding, decompress tension pneumothorax)	Control rapidly lethal etiologies.
**IN**fuse vasopressors and/or fluids	Reverse life-threatening arterial hypotension using vasopressors and/or rapid fluid bolus according to the clinical scenario and gestalt.	Achieve MAP ≥ 65 mmHg as soon as possible
**IN**vestigate main causes	Perform ECG, blood gas analysis, and send cardiac enzymes.	Conduct essential and simple tests to identify the underlying etiology.
**U**ltrasound	Conduct cardiac ultrasound to identify the type of shock. Conduct lung ultrasound to identify congestion and pneumothorax.	Use point-of-care ultrasound to identify the shock type.
**T**reat underlying **E**tiology	Specific therapy of the underlying etiology of shock (e.g., thrombolysis for pulmonary embolism, drainage of pericardial tamponade, revascularization of coronary occlusion, control of the infectious source).	Reversal of the underlying pathology causing shock.
**S**tabilize systemic organ perfusion	Evaluate (e.g., urine output, liver function, electrolytes) and stabilize systemic organ perfusion.	Optimize systemic organ perfusion and avoid fluid overload.

ECG: electrocardiogram; MAP: mean arterial blood pressure

### Recognition of shock

Accurate and timely detection is essential and unequivocally the most important step in managing patients in shock. Well-known bedside features of poor systemic perfusion are the three clinical windows: the brain (mental state), the kidneys (urine output), and most importantly the skin [10]. A recent systematic review of the literature has identified that reduced peripheral perfusion/temperature, prolonged capillary refill time, and a shock index (heart rate divided by systolic arterial blood pressure) ≥ 0.7–0.8 are valid clinical indicators of shock. It is important to note that the presence of arterial hypotension, although commonly present in patients with shock, must not be considered a prerequisite to define shock [8]. Compensatory vasoconstriction, particularly in young patients, may maintain arterial blood pressure within the normal range despite critical systemic hypoperfusion [11]. Although serum lactate level is a sensitive indicator of the presence of shock, hyperlactatemia can be rather unspecific [8]. Once shock is identified, the MINUTES bundle should be promptly implemented.

### M - Maintain “ABCs” (minute 0)

This step should be provided within the first minute of shock recognition. Providing basic and advanced life support has been well established as the initial and crucial step of resuscitation in any acutely ill patient. Therefore, ensuring airway patency, adequate ventilation, as well as presence of central pulse should be the first priorities in all patients [7, 12]. Similarly, rapidly fatal causes of shock must be identified and treated immediately (e.g., compression of external bleeding, needle-decompression of tension pneumothorax) (Table 3).

**Table 3:** Rapidly-fatal easily-detectable shock pathologies

Active external bleeding	Proper patient exposure – inspect surgical wounds	Compress bleeding site. Apply tourniquet. Pelvic binder. Replacement according to the guidelines
Tension pneumothorax	Acute severe hypoxia and hypotension Unequal chest expansion Diminished air entry Congested neck veins	Needle decompression at the 2nd intercostal space in the midclavicular line

### IN - INfuse vasopressors – INfuse fluids (minutes 0–10)

Following the ABC assessment, the mainstay of patient salvage is to restore vital organ (e.g., heart, lungs and brain) perfusion. Severe arterial hypotension can rapidly lead to myocardial hypoperfusion and death within a short period of time [1]. The term “infuse” mainly stands for infusion of vasopressors and/or fluids within a few minutes. Though fluid infusion is the first line of therapy in the initial management of shock when fluid deficit is clear, growing evidence suggests that it is safe to initiate vasopressors early in patients with septic shock in order to maintain tissue perfusion and improve venous return [13, 14]. Furthermore, excessive vasodilatation represents the most common pathophysiology of shock and requires vasopressor therapy [15]. Thus, initiation, and probably escalation, of vasopressors within the first five minutes until the etiology and type of shock have been identified is reasonable to limit the occurrence of under-perfusion and organ damage.

In case of septic shock, intravenous infusion of 30 ml/kg crystalloids has been suggested by the Surviving Sepsis Campaign within the first 3 h in patients with sepsis-induced hypoperfusion or septic shock [2]. However, fluid requirements vary substantially and such predefined volumes may result in over-resuscitation in some patients [16]. Therefore, an alternate approach has been proposed, starting with 10 mL/kg crystalloids followed by an individualized approach based on the patient response within a shorter period (one hour) than that described in the surviving sepsis campaign guidelines [17]. A similar regimen has been suggested by other authors unless clinical signs of congestion are present [13, 17]. It should be noted that fluid administration should be carefully individualized as it might be detrimental in some types of shock (e.g., cardiogenic and obstructive shock).

Whether to initiate vasopressors early or to wait for the clinical response to fluid challenge remains still debatable and likely depends on the clinical scenario and gestalt. Vasopressors may be considered early in the presence of life-threatening arterial hypotension [1], a low diastolic blood pressure < 40 mmHg, diastolic shock index > 3, or when there is a risk of fluid overload [16, 18]. It is worth mentioning that vasopressor administration could be initiated peripherally in low dilution without wasting time for central venous catheter insertion [2]. The first line vasopressor in the majority of patients with shock is norepinephrine [15].

A mean arterial blood pressure ≥ 65 mmHg appears as a suitable target during the initial salvage stage in most types of shock [2]. However, confirming adequate peripheral perfusion should not be ignored using the available indices of tissue prefusion (e.g., serum lactate, capillary refill time) [15]. In patients with hemorrhagic shock, lower targets of blood pressure values (systolic blood pressure ≈ 90 mmHg) seems more appropriate in patients without brain injury, until the source of bleeding is secured [4, 6].

### IN – Investigate (minutes 0–10)

After provision of life support and implementation of interventions to ensure vital organ perfusion, clinicians should swiftly proceed to basic investigations. Among these, VBG certainly seems one of the most appropriate for its ability to provide point-of-care results with information on several variables that may be useful for the management of shock and/or its underlying etiology. Indeed, results of serum lactate, hemoglobin, and glucose levels will provide ready-at-hand diagnostic and therapeutic support for clinicians. Point-of-care laboratory tests are widely available nowadays and can provide additional used data about electrolytes, cardiac markers, and kidney functions. Of course, other laboratory investigations (e.g., a complete blood count and, if appropriate, cardiac enzymes) may provide invaluable information, but their results are usually not available within the first hour. Finally, in all cases in whom acute myocardial ischemia cannot be excluded, clinicians should perform an electrocardiogram. We suggest that the timeframe to accomplish this step is within 10 min after shock has been recognized, ABCs maintained, and treatments for life-threatening arterial hypotension implemented.

### U – Ultrasound (minutes 10–20)

Ultrasound has several key advantages which favor its use as an initial and principal point-of-care diagnostic tool in the primary management of patients with undifferentiated shock. First, ultrasound can rapidly differentiate the pathophysiological type of shock with excellent accuracy [19], and this is particularly useful if fatal pathologies (e.g., obstructive shock) are present [10, 20, 21]. Second, ultrasound can provide useful information about fluid status (fluid responsiveness, congestion, and tolerance) whatever the type of shock is. Third, ultrasound is a cost-effective equipment which should be present in every emergency and critical care department. It is feasible in most patients without the need for expensive consumables [22]. Fourth, point-of-care ultrasound is exponentially growing [23] and has become an essential skill for all emergency and critical care physicians [24, 25]. Fifth, ultrasound has the advantage of allowing a comprehensive evaluation of several organ systems in a short time and without the need to mobilize patients, which is highly desirable under shock conditions. Accordingly, appropriate ultrasound integration in the management of patients with shock is likely to improve survival of these patients [26–28]. There is an increasing role for hand-held ultrasound which showed promising results in resource-limited settings [29, 30]. For all these reasons, we suggest early use of focused ultrasound examination as a crucial diagnostic step in the assessment of circulatory shock. It is clinically reasonable to perform this scan within the first 10–20 min after shock recognition, according to the availability of the device and presence of a skilled operator.

Once started, a focused ultrasound exam should answer the several questions. Does the patient have critical obstructive pathology? Does the patient have severe systolic dysfunction? Is there an obvious anatomical left-sided valvopathy (i.e., large vegetation)? Could the patient benefit from (or at least tolerate) a fluid bolus? These questions can be easily answered through a brief focused examination of the heart, lungs, and inferior vena cava [31]. Ultrasound can also rapidly detect intraabdominal collection, pneumothorax and some types of aortic dissection. Several protocols for point-of-care ultrasound examination are present for management of circulatory and/or respiratory failure (e.g., RUSH protocol) [32]. In case of inadequate views, ultrasound is still useful in evaluation of fluid tolerance (through examination of the lungs and inferior vena cava) and ruling our obstructive shock. More sophisticated examination steps could be considered at a later stage or be performed only by experienced echocardiographers. These may include precise measurement of the stroke volume using doppler echocardiography, fine evaluation of heart valves pathology and of diastolic function [24].

The timing of the ultrasound examination could vary according to both hospital facilities and practices. Some centers may have the resources to perform ultrasound even earlier [27]. This is likely to be beneficial by informing clinicians on the previously discussed use of fluids and/or vasopressors to reverse critical arterial hypotension. However, this practice might not be feasible in settings with limited resources. Therefore, maintaining perfusion should be the first priority whatever the timing of the ultrasound examination is. Nevertheless, once an ultrasound identifiable cause is on the top of differential diagnosis, point-of-care ultrasound should be a priority.

Several clinical signs can improve the diagnosis such was the presence of acute hypoxia (obstructive pathology), wide pulse pressure (distributive pathology), congested neck veins (obstructive pathology), and lower limb edema (cardiogenic pathology).

### TE – Treat the underlying Etiology (minutes 20–30)

Besides the pathophysiological support of circulation, treatment of the etiology of shock is the second cornerstone of shock management. Once etiology has been identified, therapeutic interventions should be directed to reverse the underlying pathology. We propose that the proper timing for this step lies after initial investigations and ultrasound, unless the primary pathology causing shock has become evident until then.

At this stage, specific management of the cause of shock should be initiated with the exception of conditions already dealt with during the early stage of the MINUTES bundle. Pulmonary embolism and cardiac tamponade are two important pathologies that should be managed appropriately in this phase. We placed these two pathologies in this phase and not earlier as their diagnosis and/or management is usually ultrasound-based [33, 34].

It should be remembered that ultrasound is not the gold standard for diagnosis of pulmonary embolism and therefore, normal cardiac ultrasound does not rule out pulmonary embolism [34]. However, according to the European guidelines, a patient with arterial hypotension and shock due to pulmonary embolism typically shows signs of right ventricular dilatation/failure. If not, other causes of shock must be considered [5]. The accuracy of point-of-care ultrasound in ruling out pulmonary embolism can be enhanced by a multiorgan approach which includes lung- and lower limb venous ultrasound examination [35].

We also highlight the importance of initiating definitive management for other causes of shock such as early antibiotic, cultures and elimination of the source in cases of sepsis [2]; hemorrhage control, transfusion and treatment of coagulopathy in patients with hemorrhagic shock [4]; as well as medical and interventional management of coronary pathologies [3].

### S – Stabilize (from minute 30 on)

This phase aims to stabilize systemic organ perfusion and continue vital organ support. The goal of the earlier phases of theMINUTES acronym was reversal of life-threatening disorders. Once the initial resuscitation goals have been achieved, physicians should rapidly move towards a more sophisticated and tailored correction of existing pathologies in order to optimize systemic organ perfusion and attenuate organ injury [15]. Among others, physicians will consider urine output, liver injury, electrolytes levels with re-evaluation and correction of residual acid-base and electrolyte disorders. Notably, this phase also includes the de-escalation of unnecessary hemodynamic and respiratory support, whenever possible. More detailed imaging such as computed tomography and advanced ultrasound examination can be performed. Indices of peripheral perfusion should be followed up to determine the patient response to resuscitation. Lactate clearance could be evaluated; however, the kinetics of serum lactate are usually slow and unlikely to reflect patient progression in a short period. Capillary refill time might be more appropriate for follow up in shorter periods [36].

Early management of shock usually includes infusion of fluid boluses unless clinical signs of congestion are present. Indeed, despite the increasing use of dynamic indices of fluid responsiveness to guide fluid administration, fluid overload is still a common problem in some patients due to capillary leak. Thus, searching for signs of fluid overload and considering subsequent strategies for the evacuation of fluids (e.g., diuresis) should be part of the clinical assessment, once the primary goals have been achieved [37, 38]. The wide use of focused ultrasound in critical care units over the last years has facilitated the chances to detect patients suffering from congestion with the evaluation of excess of extravascular lung water using lung ultrasound [37, 39]. Interestingly, a recent multicenter study found the coexistence of fluid overload signals in both fluid-responsive and non-responsive patients. This finding highlights the importance of performing a simple lung ultrasound examination in critically ill patients and this might direct the management plan towards a more fluid-conservative and vasopressor-based approach, if validated in larger studies [40].

## Conclusions

The early phases of undifferentiated shock management require a clear plan with well-defined steps, targets, and timeline. Though specific guidelines exist for the management of specific types of shock, the initial supportive plan of undifferentiated shock should be unified. We propose the MINUTES acronym to provide a simplified and simultaneously memorable timeline for the initial steps of shock management to be implemented within the first 30 min after shock recognition. The acronym includes the following components: **M**aintain “ABCs”, **IN**fuse vasopressors and/or fluids and **IN**vestigate simple blood tests, **U**ltrasound (point-of care) to detect the type of shock, **T**reat the underlying **E**tiology, and **S**tabilize systemic organ perfusion. We suggest that MINUTES would help emergency physicians to organize their management and priorities in the early critical moments of shock. Future studies are required to validate the impact of application of MINUTES on patient outcomes.

## Data Availability

No datasets were generated or analysed during the current study.
